# Efficacy of Application of Plasma Rich in Growth Factors Along with the Tunnel Technique for Treatment of Gingival Recession: a Clinical Trial

**DOI:** 10.30476/DENTJODS.2020.83590.1052

**Published:** 2020-12

**Authors:** Fatemeh Ahrari, Farshad Keshavarzi, Ali Bijani, Niloofar Jenabian

**Affiliations:** 1 Student Research Committee, Dept. of Periodontics, Faculty of Dentistry, Babol University of Medical Sciences, Babol, Iran; 2 Social Determinants of Health Research Center, Health Research Institute, Babol University of Medical Sciences, Babol, Iran; 3 Oral Health Research Center, Health Research Institute, Babol University of Medical Sciences, Babol, Iran

**Keywords:** Dental aesthetics, Surgical procedures, Gingival recession

## Abstract

**Statement of the Problem:**

The tunnel technique has shown promising results in treatment of gingival recession. Plasma rich in growth factors (PRGF) is considered effective for soft tissue regeneration since it is a rich source of growth factors.

**Purpose:**

This clinical trial aimed to assess the efficacy of PRGF along with the tunnel technique and connective tissue graft for treatment of gingival recession.

**Materials and Method:**

In this controlled clinical trial, 20 areas around anterior and posterior teeth in 3 patients with gingival recession were bilaterally selected. The tunnel technique was used with and without PRGF in the test and control groups, respectively (10 areas in each group). The keratinized gingival width (KGW), clinical attachment level (CAL), clinical probing depth (PD), cementoenamel junction (CEJ) to mucogingival junction (MGJ) distance, and the esthetic visual analog scale (EVAS) score were evaluated preoperatively and at 6 weeks and 3 months, postoperatively. The gingival recession width (RW) and vertical recession depth (VRD) were assessed preoperatively and at 2 weeks and 3 months, postoperatively. The pain visual analog scale (PVAS) score was measured at 1, 3 and 7 days, post-treatment and the healing index (HI) was measured at 1, 3 and 7 days and 1 month, postoperatively. The root coverage percentage was assessed during 3 months. Paired t-test and repeated measures ANOVA were used for statistical analyses. *p* Value< 0.05 was considered statistically significant.

**Results:**

Significant improvements were noted in all tested parameters in both groups (*p*< 0.05). The mean root coverage percentage after 6 months was 88.68%±20.69% and 78.77%±24.94% in the test and control groups, respectively. None of the tested parameters were significantly different between two groups (*p*> 0.05).

**Conclusion:**

Treatment of gingival recession with the tunnel technique can yield favorable clinical outcome, irrespective of the employment of PRGF.

## Introduction

Gingival recession is defined as apical dislodgment of the gingival margin relative to the cementoenamel junction (CEJ) due to the loss of periodontal attachments, and is currently a common clinical finding [ 1 - 2 ]. The etiology of gingival recession includes periodontal disease, poor oral hygiene, frenulum stretching, and bone dehiscence, inappropriate path of eruption of tooth, tooth malposition, gingival viral infections, and subgingival plaque accumulation [ 3 ]. In addition, tooth-brushing trauma, especially in young individuals, can play a role in progression of gingival recession [ 4 ]. Gingival recession can compromise smile esthetics and affect the mastication function as well [ 5 - 6 ]. Also, it complicates plaque control [ 4 ]. Due to the complications of gingival recession such as tooth hyper-sensitivity [ 7 ], root caries [ 8 ], esthetic concerns and decreased attached gingiva, periodontists have long been in search of novel techniques for proper management of this condition. Treatment of gingival recession is highly challenging for dental clinicians since an ideal treatment for gingival recession should be able to restore the lost anatomy of the mucogingival junction (MGJ), improve the esthetic appearance of the tooth, regenerate or restore cementum, induce reattachment of periodontal fibers and generation of the supporting bone, eliminate tooth hyper-sensitivity and prevent root caries. To date, several surgical techniques such as laterally positioned flap [ 9 ], coronally positioned flap [ 10 ], free gingival graft [ 11 - 12 ] and sub-epithelial connective tissue graft with coronally positioned flap [ 13 ] have been introduced and evaluated for treatment of gingival recession. However, application of coronally advanced flap along with connective tissue graft (bilaminar technique) currently serves as the gold standard for this purpose [ 14 ]. Since adequate keratinized gingiva is not available at the recession site, soft tissue grafts are obtained from an intraoral donor site. The use of connective tissue for root surface coverage was first suggested by Langer and Calagna [ 15 ] in 1982. In graft procedures, the main challenge is to preserve the blood supply of the recipient's site to avoid necrosis and defects. Palatal mucosa can serve as an appropriate graft donor site due to its keratinized tissue [ 15 - 16 ]. 

Therefore, an intraoral autogenous graft with proper blood supply can be helpful for treatment of gingival recessions. The tunnel technique as a one-step surgical procedure can be used for treatment of Miller’s class 1 and 2 multiple gingival recessions [ 16 ]. The length of the hard palate and the thickness of the palatal mucosa are important factors to consider in selection of this technique. This technique has many advantages. It preserves the integrity of the papilla, and the flap does not require a vertical releasing incision; thus, the connective tissue and periosteal hemorrhage is prevented. The surgery is performed with minimal trauma. Also, the grafted tissue is fixed and does not move in this technique and is also less exposed. The level of pain of patients is often minimal after this procedure and the healing period is fast. Moreover, esthetic results are achieved sooner than other techniques with minimal scarring. 

According to the protocol described by Allen [ 17 ], the tunnel technique requires a supra-periosteal mucosal flap with intra-sulcular incisions. By using this method, cervical gingival movement can be achieved by creating a space. Next, the interdental papilla is undermined and a mucogingival tunnel is created between the spaces. After that, a connective tissue graft is placed inside the tunnel, with part of it covering the recessed gingiva. It is then sutured in a fixed position [ 4 ]. 

The use of endogenous and biologically active proteins for regenerative purposes has opened a new path for tissue regeneration. Growth factors are biological mediators that play a key role in proliferation, chemotaxis, and cell differentiation. They act through specific receptors located on the surface of the cells and guide the healing process. Growth factors are like hormones that are not released into the blood circulation and only act locally; some growth factors can cause premature changes in the G0 to G1 phases of cell division, and even have the ability to stimulate DNA synthesis in some certain cells [ 12 ]. 

In 1999, Anitua [ 18 ] described a new technique for preparation of plasma-rich platelets known as plasma rich in growth factors (PRGF). PRGF is prepared autogenously and is rich in biological mediators that accelerate hard and soft tissue regeneration. Plasma-derived adhesion molecules such as fibrinogen, fibronectin, vitronectin and thrombospondin-1 act as a matrix or scaffold and attract precursor cells and platelets. Platelets are a rich source of growth factors such as platelet- derived growth factor, transforming growth factor beta, vascular endothelial growth factor, fibroblast growth factor, insulin-like growth factor and granulocyte-macrophage colony-stimulating factor [ 19 ].

Search of the literature by the authors yielded no study on the application of PRGF with the connective tissue graft and the tunnel technique for treatment of gingival recessions. Thus, further investigations are required on this topic. Also, it has been hypothesized that addition of PRGF to the connective tissue graft may be able to increase the success rate of the tunnel technique close to that of the gold standard. This hypothesis is also in need of further investigation. 

Considering the advantages of PRGF, this clinical trial aimed to evaluate the efficacy of the tunnel technique and connective tissue graft in combination with PRGF for treatment of gingival recession by assessing its effect on gingival parameters.

## Materials and Method

In this controlled clinical trial, 20 areas around anterior and posterior teeth (lateral incisors, canine, first premolar and second premolar teeth) in 3 patients with gingival recession were bilaterally selected. The tunnel technique was used with and without PRGF in the test and control groups, respectively (10 areas in each group). The keratinized gingival width (KGW), clinical attachment level (CAL), clinical probing depth (PD), CEJ to MGJ distance and the esthetic visual analog scale (EVAS) score were evaluated before treatment and at 6 weeks and 3 months after treatment. The gingival recession width (RW) and vertical recession depth (VRD) were evaluated at baseline and at 2 weeks and 3 months after the treatment. The pain visual analog scale (PVAS) score was also evaluated at 1, 3 and 7 days, after surgery. The healing index (HI) was evaluated at 1, 3 and 7 days, and 1 month after treatment. The root coverage percentage was evaluated during the 3-month study period.

KGW was characterized as the distance between the free gingival margin and the MGJ. The CAL was defined as the distance from the CEJ to the bottom of the gingival margin at the midpoint of the buccal gingival margin. PD was the distance between the free gingival margin and the bottom of the gingival sulcus at the midpoint of the buccal gingival margin [ 20 ]. VRD was the distance between the CEJ and the free gingival margin (at the midpoint of the buccal surface). The RW was defined as the width of recession at 1 mm apical to the CEJ in the mesiodistal dimension. The CEJ to MGJ distance was the distance between the CEJ and MGJ in the middle of the buccal surface of the tooth [ 21 ]. In order to assess the possible movement of the MGJ, CEJ was considered as a fixed reference line. The root coverage percentage was calculated based on the following equation:

$\frac{\mathrm{Vertical depth of gingival recession before surgery-(Vertical depth of gingival recession after surgery)}}{\mathrm{Vertical depth of gingival recession before surgery}}\times 100$

The HI was calculated according to the criteria by Landry [ 22 ]. The EVAS score was determined using a 0-10 scale where 0 is the most unpleasant esthetic appearance according to the patient’s point of view while 10 is the most pleasant appearance. The PVAS score was determined using a 0-10 scale with 10 showing maximum pain and 0 indicating no pain at all. 

The inclusion criteria were a minimum of 18 years of age, having Miller’s class I/II single, facial, bilateral gingival recessions with ≥2 mm depth from the CEJ around the anterior and posterior vital teeth (lateral incisors, canine, first premolar and second premolar teeth) with no restoration and no bleeding on probing, patient's ability to maintain proper oral hygiene (O’Leary’s plaque score ≤ 20%), KGW greater than 2 mm and gingival thickness ≥ 0.5 mm (2 mm apical to the gingival margin). The exclusion criteria were pregnancy, coagulation disorders, use of medications that interfere with platelet function (non-steroidal anti-inflammatory drugs) or wound healing (corticosteroids, anticancer medications), local or systemic diseases contraindicating periodontal treatment, history of allergy to surgical materials, active infectious diseases (hepatitis, tuberculosis or AIDS), smoking, frenum pull at the surgical site, traumatic tooth brushing, and use of removable appliances and prosthesis in the area.

**Figure 1:**
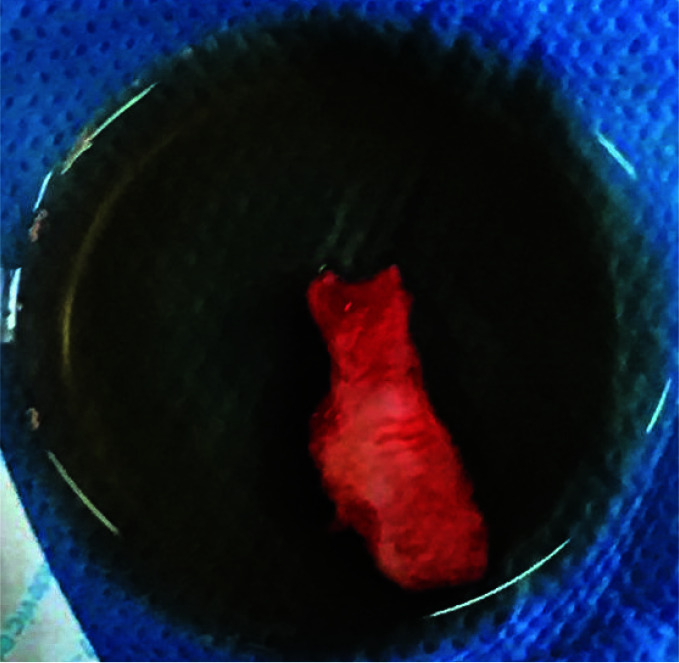
PRGF-impregnated connective tissue

The study was approved by the Ethics Committee of Babol University of Medical Sciences on February 26, 2018 (MUBABOL.HRI.REC.1396.210). The study was also registered in the Iranian Registry of Clinical Trials (IRCT20100427003813N9). All patients received phase 1 periodontal therapy before the surgical procedure and signed informed consent forms (Figure 1 [Fig JDS-21-275-g003.tif][Fig JDS-21-275-g004.tif]to 5). The surgical areas were randomly divided into two groups. 

**Figure 2:**
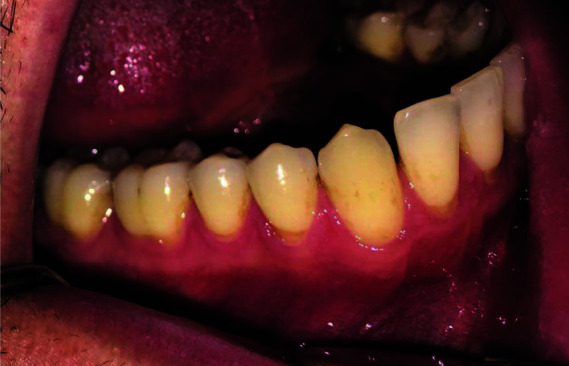
Preoperative intraoral view

**Figure 3 JDS-21-275-g003.tif:**
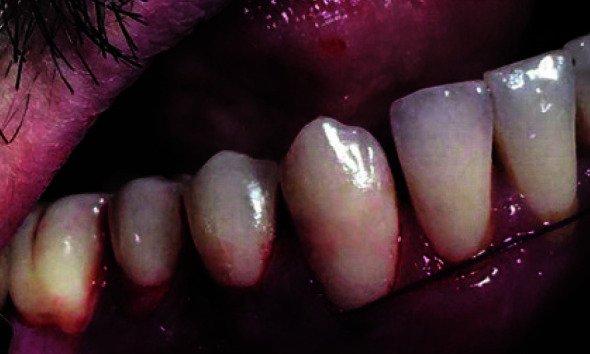
Intraoral view during surgery

**Figure 4 JDS-21-275-g004.tif:**
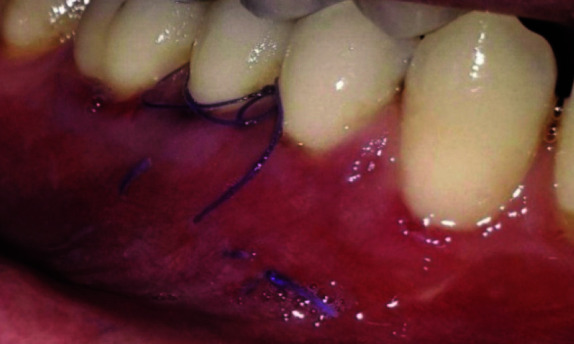
Intraoral view 2 weeks after surgery

**Figure 5:**
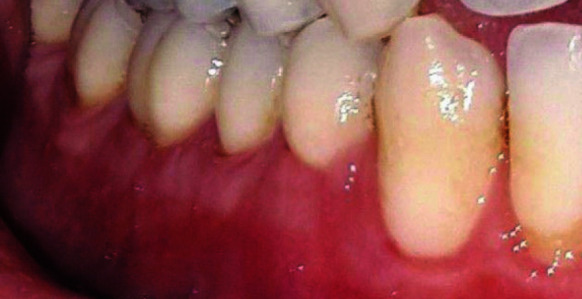
Intraoral view 3 months after surgery

In one side, the gingival recession was treated with the tunnel technique using PRGF (test group) while on the other side, gingival recession was treated with the tunnel technique without PRGF (control group). All surgical procedures were performed by one single surgeon. In the preoperative phase, the clinical parameters were measured by an examiner who was blinded to the procedure and group allocation of sites. The measurements were made using a periodontal probe (Williams probe; HU-Friedy, Chicago, IL, USA) with 1mm accuracy. PRGF was prepared right before surgery as described by Anitua [ 23 ]. Before surgery, 20 mL of venous blood was collected from the patient and transferred into 5 mL tubes containing 3.8% sodium citrate as anticoagulant. Then, the tubes were centrifuged (Endoret Technology system IV, the BTI Biotechnology institute, Minano, Alva, Spain) at room temperature for 8 minutes. After centrifugation, the contents of each tube included: (1) a plasma layer (1 mL) formed on the top which contained a small amount of growth factors or plasma poor in growth factor (PPGF), (2) a second plasma layer with 0.5 mL volume containing two times the growth factor concentrate or plasma with growth factor (PGF), (3) PRGF (0.5mL) between the second layer and the white blood cell layer, (4) a white layer (50µL) of white blood cells between the PRGF and the red blood cells, and (5) The red blood cells (RBC) layer. The first and the second layers were separated and transferred into separate tubes using 500μL pipettes. For more precision and in order to prevent any disturbances between the PRGF layer and the white blood cell layer, the third layer was gently removed using a 100-μL pipette 5 times and transferred into another tube. Then, activation was done by adding 50μL of 10% calcium chloride per each 1mL of PRGF.

This study was performed by two researchers with following steps. The grafting procedure was carried out by the first practitioner according to the instructions, such that after local anesthesia administration with 2% lidocaine plus 1:80,000 epinephrine by the infiltration technique, root planing was performed with Gracey curettes. Primary sulcular incisions were made using a 15C blade, and an intra-sulcular incision was made on the buccal surface around the tooth neck. This incision was extended to the mesial and distal by one tooth. The papilla remained intact and was just undermined and gently separated from the bone. A tunnel full-thickness envelope was created and extended beyond the mucogingival line. In the test group, the connective tissue obtained from the palatal area of ​​the surgical site (a class II type A incision according to the Liu’s classification) was shaped and immediately impregnated with PRGF before its conversion to gel form (it was only added to the graft and not the root surface). Next, it was applied on the surface of the root (root planning). In the control group, the impregnation was not performed. The thickness of connective tissue graft was considered to be 1.5mm in all patients (standardized by a caliper). The graft was stabilized by absorbable 5-0 sutures using the vertical mattress technique. Finally, the flap was positioned coronally using a horizontal mattress suture that was anchored to the tooth. In addition to daily oral hygiene, the patients were instructed to rinse their mouth with 0.12% chlorhexidine gluconate mouthwash twice a day for 4 weeks [ 24 ]. Ibuprofen (400mg) was administered 3 times daily for 7 days and 500 mg amoxicillin was prescribed 3 times daily for 10 days [ 21 ]. Periodic examinations were performed by the second researcher who was unaware of the surgical technique. Plaque control was performed periodically. At 1 month and 3 months, patients were recalled to measure their clinical parameters [ 21 , 24 ]. 

Statistical analyses were performed using SPSS version 20 software. Changes in the variables were measured at the aforementioned time points. The mean and standard deviation of all clinical variables in each group were reported. The Kolmogorov-Smirnov test was used to assess the normal distribution of data. Within-group and between-group differences were analyzed before and after treatment using t-test and paired t-test. Comparisons were carried out using repeated measures ANOVA (non-parametric test) whenever required. *p*< 0.05 was considered statistically significant.

## Results

Both groups showed significant changes in KGW at 6 weeks (*p*< 0.001 in the test and *p*< 0.001 in the control group) and 3 months (*p*< 0.001 in the test and *p*< 0.001 in the control group) after treatment compared with baseline. The changes in CAL were also significant at 6 weeks (*p*< 0.001 in the test and *p*< 0.001 in the control group) and 3 months (*p*< 0.001 in the test and *P* < 0.001 in the control group) after surgery compared with baseline in both groups (Table 1). The RW showed significant changes after 2 weeks and 3 months compared with baseline (*p*< 0.001 in the test and *p*< 0.001 in the control group) (Table 2). Furthermore, VRD experienced a significant change after 2 weeks and 3 months compared with baseline (*p*< 0.001 in the test and *p*< 0.001 in the control group) (Table 2).

**Table 1:** Changes in keratinized gingival width (KGW), clinical attachment level (CAL), clinical probing depth (PD), CEJ to MGJ distance (MGL_L_), esthetic index (E_VAS_), gingival recession width (RW), and vertical recession depth (VRD)

Index	Group	Baseline	6 weeks after treatment	3 months after treatment	*p* Value
KGW	Experimental	3.60 ± 0.669	5.35 ± 0.668	5.20 ± 0.752	<0.001
	Control	3.60 ± 0.699	5.80 ± 1.22	5.55 ± 1.06	<0.001
	p	1.000	0.323	0.407	0.399 a
CAL	Experimental	3.10 ± 0.994	1.350 ± 0.851	1.350 ± 0.851	<0.001
	Control	3.00 ± 0.408	1.150 ± 0.818	1.20 ± 0.919	<0.001
	P	0.772	0.599	0.709	0.881 a
PD	Experimental	0.700 ± 0.258	0.700 ± 0.258	0.700 ± 0.258	0.564
	Control	0.650 ± 0.241	0.600 ± 0.210	0.650 ± 0.242	1.000
	P	0.660	0.355	0.660	0.913 a
MGLL	Experimental	5.85 ± 0.747	7.50 ± 1.00	7.50 ± 0.84	<0.001
	Control	5.700 ± 0.586	7.60 ± 1.39	7.60 ± 1.34	<0.001
	P	0.624	0.856	0.845	0.650 a
EVAS	Experimental	5.00 ± 0.666	9.00 ± 1.33	9.00 ± 1.33	<0.001
	Control	5.00 ± 0.667	9.00 ± 1.33	9.00 ± 1.33	<0.001
	p	1.000	1.000	1.000	1.000 a

a The comparison of trend and group

**Table 2:** Changes in gingival recession width (RW), and vertical recession depth (VRD)

Index	Group	Baseline	2 weeks after treatment	3 months after treatment	*p* Value
RW	Experimental	3.40 ± 0.567	0.800 ± 1.03	0.600 ± 1.07	<0.001
	Control	3.40 ± 0.459	0.750 ± 1.08	0.350 ± 0.747	<0.001
	P value	1.000	0.617	0.553	0.751 a
VRD	Experimental	2.20 ± 1.159	0.450 ± 0.955	0.450 ± 0.955	<0.001
	Control	2.40 ± 0.459	0.600 ± 0.738	0.550 ± 0.643	<0.001
	P value	0.618	0.695	0.787	0.881^a^

a The comparison of trend and group

The CEJ to MGJ distance was significantly different at baseline, and at 6 weeks and 3 months after surgery in both groups (*p*< 0.001 in the test and *p*< 0.001 in the control group). The test group did not show any significant difference with the control group regarding the percentage of root coverage, which was investigated during a three-month period (p= 0.347). The average percentage of root coverage after 6 months was 88.68% ± 20.69% and 78.77%±24.94% in the test and control groups, respectively. Table 1 presents the results regarding PVAS score, HI, PD and EVAS score. All measured parameters showed significant improvement after treatment in both groups. But, none of the evaluated parameters were significantly different between the two groups (*p*> 0.05). 

No significant difference was noted in HI between the two groups (p= 0.322) at any time point. The difference in PVAS score was not significant between the two groups at any time point (p= 0.984). 

## Discussion

According to the results of this study, although there was a significant improvement after treatment in both groups, there were no significant differences between the two groups in any parameter. The results of clinical studies about PRGF are slightly different from in vitro results. According to an in vitro study by Anitua *et al*. [ 18 ], use of PRGF significantly increased the proliferation and migration of gingival fibroblasts, and their attachment to type 1 collagen matrix. It also stimulated the expression of autocrine endothelial growth factor, hepatocyte growth factor, and hyaluronic acid; consequently, it was reported to be effective for periodontal regeneration. Thus, PRGF is expected to enhance the treatment of gingival recession [ 19 ]. However, the results of previous clinical studies regarding the treatment of gingival recessions with different techniques with and without PRGF are different from our findings. The results of this study are in accordance with those of Jenabian *et al*. [ 21 ], who used PRGF with connective tissue grafts for treatment of gingival recession and showed no significant difference between groups. Abolfazli *et al*. [ 25 ] investigated the efficacy of double pedicle graft with and without PRGF in treatment of class 1 and 2 gingival recessions. The clinical parameters such as clinical PD, CAL, RW, and KGW were evaluated at the onset of treatment and at 1, 3 and 6 months after treatment. The authors showed that despite significant improvements in the depth and width of recession as well as the KGW in both groups, the differences between the groups were not significant. Lafzi *et al*. [ 26 ] used coronally advanced flap with and without PRGF and showed that although PRGF caused a significant difference in the first month, the difference between the groups was no longer significant in the second month. They used the switch technique with and without PRGF and showed that PRGF improved the results of coronally advanced flap during the first month after surgery, but the indices did not differ significantly in the last two months of periodic examinations. Although different procedures were utilized, the results regarding the inefficacy or low efficacy of PRGF were the same. 

Our results regarding PD were in accordance with those of Keceli *et al*. [ 1 ], Lafzi *et al*. [ 26 ], and Jankovic *et al*. [ 27 ] who found no significant change during the study period. Differences in PD values may be attributed to differences in techniques employed and differences in the baseline values of PD in different researches, making it intricate to contrast the results.

In this study, the mean reduction of VRD in the two groups (2.20 mm in the test and 2.40mm in the control group) showed greater changes in comparison with the studies by Jenabian *et al*. [ 21 ] (1.36mm and 0.95mm) and Huang *et al*. [ 28 ]; however, it was similar to the study by Thalmair *et al*. [ 29 ] (2.7mm). The reason for this finding may be related to the different techniques used in the studies, because Jenabian *et al*. [ 21 ] and Huang *et al*. [ 28 ] used subepithelial connective tissue graft technique; while, in the present study and the study by Thalmair *et al*. [ 29 ] the tunnel technique was adopted to treat gingival recession. It seems that the tunnel technique is more predictable than the subepithelial connective tissue graft technique, which may be due to its lower invasiveness and higher blood supply. The baseline KGW value may be another reason for different findings of studies, as in the study by Jenabian *et al*. [ 21 ] the pre-treatment KGW was recorded to be 4.8mm and 4mm in the case and control groups, respectively, while in our study, this value was 3.6 mm. The same result was found in the study by Lafzi *et al*. [ 30 ].

In the current study, the mean percentage of root coverage in the test and control groups was 88.6% and 78.7%, respectively in a three-month period. This index was respectively 80.3% and 67.4% in the study of Jenabian *et al*. [ 21 ]. The findings of other studies such as those of Lafzi *et al*. [ 26 ], Jankovic *et al*. [ 27 ], and Cheung *et al*. [ 31 ] were similar to the results of Jenabian *et al*. [ 21 ]. However, in the present study, the mean percentage of root coverage was higher than that reported by previous studies [ 26 - 27 , 32 ]. Again, difference in technique of treatment seems to play a role in controversial results. 

KGW is an important parameter when interpreting the outcome of treatment of gingival recession. It is also important in maintaining gingival health [ 21 ]. In the present study, the mean KGW in the experimental group increased by 1.75 mm and 1.6 mm at 6 weeks and 3 months after treatment, respectively. This value was 2.2 mm and 1.95 mm, respectively in the control group, and there was no statistically significant difference between the two groups. These values in the study by Jenabian *et al*. [ 21 ] were lower at 6 months (0.95 and 0.59 mm, respectively). Also, other studies indicated different magnitudes of improvement in KGW [ 28 , 31 , 33 - 34 ]. 

The pain score in the present study was evaluated using the VAS. In general, the mean pain score in the test group was significantly lower than that in the control group. Also, in the study by Jankovic *et al*. [ 27 ] the severity of pain in the connective tissue graft group was significantly higher than that in platelet-rich plasma group with connective tissue graft. Moreover, in both groups, the level of pain significantly decreased in the first 7 days. In the study by Jenabian *et al*. [ 21 ], there was no significant difference in pain score between the two groups. Differences in pain outcome may be due to different surgical techniques, post-surgical medications, surgeons’ skills, and the threshold of pain in patients.

In this study, we quantified the tissue repair using the HI by Landry [ 22 ]. The results showed that tissue repair increased from day 1 after treatment to 1 month after treatment, and recovery was achieved, but the difference in tissue repair between the two groups was not significant. In the study by Jenabian *et al*. [ 21 ], there was no significant difference in tissue repair between the experimental and control groups; however, in the study by Jankovic *et al*. [ 27 ] wound healing was better in the group with platelet-rich plasma than the group with connective tissue graft alone. This difference may be related to the different methodologies. 

In the present study, the mean CAL significantly decreased in both groups (1.75mm in the test and 1.8mm in the control group) by up to 3 months after the treatment; but the difference in this regard was not significant between the study groups. This finding was similar to the results of Jenabian *et al*. [ 21 ], who showed that the mean CAL at 6 months after treatment significantly decreased in both groups (23.1 mm in the experimental group and 1 mm in the control group). However, the difference between the groups was not significant.

In this study, the RW significantly decreased in both groups (2.8 mm in the test and 3.25 mm in the control group after 3 months). However, the difference was not significant between the groups. In general, the mean distance between CEJ and MGJ increased at 3 months after treatment, but there was no significant difference between the two groups. Jenabian *et al*. [ 21 ] found similar results and did not report significant differences between the two groups either.

In this study, the esthetic index in both groups did not differ significantly at any time point. This finding was in contrast to the results of some studies. Jenabian *et al*. [ 21 ] observed better results in terms of esthetics in the group that received connective tissue graft alone. Cheung and Griffin [ 31 ] reported better results in the PRGF group. The reason for the difference in results may be related to different concepts of beauty among different individuals [ 35 ]. For instance, in the study by Cheung and Griffin [ 31 ], a periodontist assessed the color, consistency, and contour of gingiva while in the present study; the patients determined EVAS score by themselves. Although Jenabian *et al*. [ 21 ] calculated the EVAS score; they followed up the patients for up to 6 months after treatment. In the current study, patients were evaluated for a maximum of 3 months after treatment, and the time factor could have had a significant effect on the outcome and may be responsible for the difference in the results.

In general, there were no significant differences between the two groups in the tested parameters. This result suggests that in the tunnel technique, the use of PRGF does not make a significant difference in gingival parameters. Khuller *et al*. [ 32 ] who evaluated the amount of gingival coverage using connective tissue graft with the tunnel technique showed that the tunnel technique has many advantages and is effective in treatment of gingival recession. The finding of current study agrees with their study. The study of Calin *et al*. [ 4 ] evaluated the tunnel technique and reported that this method accelerates the healing process and leads to more esthetic results considering the minimally invasive nature of this approach and the significant improvement in gingival parameters in periodic examinations. They concluded that this technique would be more easily accepted by patients and results in higher patient satisfaction. Thalmair *et al*. [ 29 ] in a prospective clinical trial on patients with gingival recession using the tunnel technique and gingival connective tissue graft showed that based on the VRD, the PD and the KGW, the tunnel technique has a high success rate in gingival coverage of recessed gingiva. The reason for these findings may be related to maintaining the vascular base and blood supply of graft tissues in this technique.

One limitation of this study was the small sample size; however, the number of areas evaluated was adequate according to the statistical equation that calculates the sample size. The patients were not significantly different in terms of type of recession and response to treatment. According to this study, although use of PRGF in conjunction with the tunnel technique was not much effective in treatment of gingival recession, further studies with larger sample size and longer follow-ups are still required to convey a final verdict in this respect. 

## Conclusion

The current results indicated that treatment of gingival recession with the tunnel technique, irrespective of the use or no use of PRGF would yield optimal clinical results. Application of PRGF had no significant effect on the results. The prospective studies should reflect on longer follow-up periods and larger sample size in conjunction with histological evaluations in order to evaluate the effect of PRGF on re-establishment of periodontal attachments.
